# The frequent exacerbator phenotype in bronchiectasis revisited: data from EMBARC registry

**DOI:** 10.1093/ajrccm/aamag297

**Published:** 2026-06-25

**Authors:** Oriol Sibila, Lidia Perea, Pierre Regis Burgel, Eva Polverino, Rebecca C Hull, Merete B Long, Sanjay H Chotirmall, Michal Shteinberg, Jennifer Pollock, Jamie Stobo, Holly Lind, Charles Haworth, Katerina Dimakou, Apostolos Bossios, Michael R Loebinger, Raja Dhar, Jin-Fu Xu, Pontus Mersch, Jessica Rademacher, Hayoung Choi, Natalie Lorent, Francesco Blasi, Montserrat Vendrell, Pieter Goeminne, Felix Ringshausen, Anthony De Soyza, Stefano Aliberti, James D Chalmers

**Affiliations:** Respiratory Department, Hospital Clinic, University of Barcelona, IDIBAPS, CIBERES, Barcelona, Spain; Institut d’Investigacions Biomèdiques August Pi i Sunyer (IDIBAPS), Research Group of Inflammation and Repair in Respiratory Diseases, Barcelona, Spain; Department of Respiratory Medicine and French Cystic Fibrosis National Reference Center, Hôpital Cochin, AP-HP and Université Paris Cité, Inserm U1016, Institut Cochin, Paris, France; Pneumology Department, Hospital Universitari Vall d‘Hebron, Vall d’Hebron Institut de Recerca (VHIR), Vall d’Hebron Barcelona Hospital Campus, CIBERES, Barcelona, Spain; Division of Molecular and Clinical Medicine, University of Dundee, Ninewells Hospital and Medical School, Dundee, United Kingdom; Division of Molecular and Clinical Medicine, University of Dundee, Ninewells Hospital and Medical School, Dundee, United Kingdom; Lee Kong Chian School of Medicine, Nanyang Technological University, Singapore, Singapore; Department of Respiratory and Critical Care Medicine, Tan Tock Seng Hospital, Singapore, Singapore; Pulmonology Institute and CF Center, Carmel Medical Center, Haifa, Israel; B. Rappaport Faculty of Medicine, Technion-Israel Institute of Technology, Haifa, Israel; Division of Molecular and Clinical Medicine, University of Dundee, Ninewells Hospital and Medical School, Dundee, United Kingdom; Division of Molecular and Clinical Medicine, University of Dundee, Ninewells Hospital and Medical School, Dundee, United Kingdom; Division of Molecular and Clinical Medicine, University of Dundee, Ninewells Hospital and Medical School, Dundee, United Kingdom; Royal Papworth Hospital NHS Foundation Trust and University of Cambridge, Cambridge, United Kingdom; ATTICA Rehabilitation Center, Athens, Greece; Department of Respiratory Medicine and Allergy, Karolinska University Hospital, Stockholm, Sweden; Division of Lung and Airway Research, Institute of Environmental Medicine, Karolinska Institute, Stockholm, Sweden; Host Defence Unit, Royal Brompton Hospital, National Heart and Lung Institute, Imperial College, London, United Kingdom; Department of Pulmonology, CK Birla Hospitals, Kolkata, India; Department of Respiratory and Critical Care Medicine, Tongji Hospital, Tongji University, Shanghai, China; Department of Medicine V, University Hospital, LMU Munich, Comprehensive Pneumology Center Munich, Member of the German Center for Lung Research, Munich, Germany; Department of Respiratory Medicine and Infectious Diseases, Hannover Medical School, Hannover, Germany; Biomedical Research in End-Stage and Obstructive Lung Disease Hannover, German Centre for Lung Research, Hannover, Germany; Division of Pulmonary, Allergy, and Critical Care Medicine, Department of Internal Medicine, Hallym University Kangnam Sacred Heart Hospital, Seoul, Republic of Korea; Department of Respiratory Diseases, University Hospitals Leuven, Leuven, Belgium; Department of Chronic Diseases, Metabolism and Aging, BREATHE Laboratory, KU Leuven, Leuven, Belgium; Department of Pathophysiology and Transplantation, University of Milan, Milan, Italy; Fondazione Istituto di Ricovero e Cura a Carattere Scientifico Ca’ Granda Ospedale Maggiore Policlinico, Milan, Italy; Department of Pulmonology, Dr Trueta University Hospital, Girona Biomedical Research Institute, Cerca. University of Girona, Girona, Spain; Department of Respiratory Disease, AZ Nikolaas, Sint-Niklaas, Belgium; Department of Respiratory Medicine and Infectious Diseases, Hannover Medical School, Hannover, Germany; Biomedical Research in End-Stage and Obstructive Lung Disease Hannover, German Centre for Lung Research, Hannover, Germany; European Reference Network on Rare and Complex Respiratory Diseases, Frankfurt am Main, Germany; Population and Health Sciences Institute, NIHR Biomedical Research Centre for Aging, Newcastle University and Department of Respiratory Medicine, Newcastle upon Tyne NHS Foundation Trust, Newcastle upon Tyne, United Kingdom; Department of Biomedical Sciences, Humanitas University, Milan 20072, Italy; Respiratory Unit, IRCCS Humanitas Research Hospital, Milan 20089, Italy; Division of Molecular and Clinical Medicine, University of Dundee, Ninewells Hospital and Medical School, Dundee, United Kingdom; Radcliffe Department of Medicine, University of Oxford, Oxford, United Kingdom

**Keywords:** exacerbation risk, etiology, region

## Abstract

**Rationale:**

The frequent exacerbator phenotype was previously defined using a threshold of  ≥3 exacerbations per year in bronchiectasis. However, the contribution of each prior exacerbation to future risk as well as the influence of severe exacerbations and different etiologies and regions remain poorly understood.

**Objectives:**

To quantify the risk associated with each prior exacerbation of bronchiectasis in predicting future exacerbations and severe exacerbations, and to determine whether this association varies across different etiologies and geographic regions.

**Methods:**

We analyzed data from the European Bronchiectasis Registry (EMBARC), including 30 countries across Europe and Asia. The association between baseline exacerbation history and future exacerbations was tested using negative binomial regression over up to 5 years of follow-up. Severe exacerbations were defined as those requiring hospitalization.

**Measurements and Main Results:**

A total of 19 324 patients with bronchiectasis were included. Each prior exacerbation was associated with an increased risk of exacerbations and severe exacerbations. Incidence rate ratio (IRR) for future exacerbations was 1.45 (95% CI 1.34-1.58, *P* < .001) for 1 exacerbation; 1.84 (95% CI 1.69-1.99, *P* < .001) for 2 exacerbations; 2.50 (95% CI 2.29-2.73, *P* < .001) for 3 exacerbations; and 3.56 (95% CI 3.32-3.82, *P* < .001) for patients with 4 or more prior exacerbations. Additionally, 1 prior severe exacerbation was associated with increased risk of exacerbation (IRR = 1.52, 95% CI 1.45-1.60, *P* < .001) and strongly associated with future severe exacerbations (IRR = 3.96, 95% CI 3.71-4.22, *P* < .001). The frequent exacerbator phenotype was consistent across different etiologies and geographic regions.

**Conclusions:**

The frequent exacerbator phenotype is highly consistent across patient subgroups and regions. Each prior exacerbation is associated with a progressively higher risk, with no definitive threshold-defining high risk.

At a Glance Commentary **Scientific Knowledge on the Subject:** Exacerbations are a key driver of disease progression in bronchiectasis, and prior exacerbation history is used in clinical guidelines to identify patients at increased risk and guide treatment decisions. The frequent exacerbator phenotype has traditionally been defined using a threshold of ≥3 exacerbations per year. However, the relative contribution of each prior exacerbation, including severe exacerbations requiring hospitalization, and whether this relationship is consistent across different etiologies and geographic regions remain poorly defined.

**What This Study Adds to the Field:** In a large international cohort from the EMBARC registry including more than 19 000 patients, each prior exacerbation was associated with a progressively higher risk of future exacerbations, demonstrating a graded risk rather than a simple threshold effect. Severe exacerbations further increased this risk, and were strongly associated with severe exacerbations. The association between prior and future exacerbations was consistent across bronchiectasis etiologies and geographic regions, supporting the frequent exacerbator phenotype as a robust and globally applicable clinical concept.

## Introduction

Bronchiectasis is a chronic respiratory disease characterized by irreversible bronchial dilatation, persistent airway inflammation, impaired mucociliary clearance and recurrent respiratory infections, ultimately leading to progressive structural lung damage.^1^^,^^2^ Beyond this unifying definition, bronchiectasis is highly heterogeneous and has multiple causes, different microbiology, and varying severity and response to treatment.^3^^,^^4^

Exacerbations can predict disease progression in bronchiectasis because they are associated with lung function decline, worsening structural lung damage, increased healthcare resources, and higher morbidity and mortality.^5^^,^^6^ Data from the European Bronchiectasis Registry (EMBARC) and other international registries suggested that up to one-third of patients experienced 3 or more exacerbations per year, a threshold previously used to define the “frequent exacerbator phenotype”^7^^,^^8^, which is associated with worse clinical outcomes such as worse quality of life (QoL) and increased mortality risk.^9^

Prior exacerbations have been recognized as the major predictor of future exacerbations, and current international clinical guidelines use prior exacerbations to identify patients at risk and guide pharmacological treatments.^10^ A key novel concept introduced by the new 2025 European Respiratory Society (ERS) clinical practice guideline is the individualized risk assessment. Long-term treatments with macrolides and/or inhaled antibiotics are recommended for patients with 2 or more exacerbations per year, with at least 1 severe exacerbation requiring hospitalization per year, or with at least 1 exacerbation per year in the presence of severe symptoms.^10^ This is an important difference to previous ERS and national guidelines, when prophylactic treatment was only recommended for those patients with 3 or more prior exacerbations.^11^^,^^12^ However, the contribution of each prior exacerbation, as well as the importance of prior severe exacerbations in predicting future risk, is not well defined, making these new thresholds controversial.

Bronchiectasis is highly heterogeneous between different regions with clear differences in patient characteristics and outcomes between patients in Eastern Europe and Asia, compared to Western European countries where the majority of prior research studies have originated.^6^^,^^13^ Different studies have demonstrated an increased risk of exacerbations in patients with bronchiectasis related to chronic obstructive pulmonary disease (COPD), primary ciliary dyskinesia (PCD), or allergic bronchopulmonary aspergillosis (ABPA).^14-16^ Moreover, the presence of chronic bacterial infection, mainly caused by *Pseudomonas aeruginosa*, also has been related to increased exacerbation risk,^17^^,^^18^ and it is well known that microbiology patterns differ among regions.^7^ Recent studies using next-generation shotgun metagenomics sequencing confirm and extend previous observations of regional variation in bronchiectasis microbiology, including variation in the distribution of pathogens and antimicrobial resistance.^8^^,^^19^

We hypothesized that each prior exacerbation, including severe exacerbations requiring hospitalization, would be associated with an increased risk of future exacerbations and severe exacerbations, and that factors such as etiology or geographical region might influence the relationship between prior and future exacerbations in patients with bronchiectasis. Therefore, we aimed to quantify the risk associated with each prior exacerbation, including severe exacerbations, in predicting future exacerbations, and to determine whether this association varied across different etiologies and regions. For that purpose, we used data from the European Bronchiectasis Registry (EMBARC), a large international registry for adults with bronchiectasis.

## Methods

### Study design

The European Bronchiectasis Registry (EMBARC) is an observational study of patients with computed tomography confirmed bronchiectasis conducted across 30 countries.^7^^,^^20^ The registry includes European and Asian countries. The study received central ethical approval from the Multicenter Research Ethics Committee in the UK (host country) on January 8, 2015, (14/SS/1101) and by institutional review boards or ethics committees in all countries and regions in which the study is conducted. All patients signed an informed consent. A detailed protocol of the study and baseline patient characteristics have been previously published.^7^^,^^20^

Patients enrolled between January 2015 and April 2022 were included in this analysis. Patient data were collected annually using a standardized case report form. Comprehensive clinical data incorporating demographics, comorbidities, medications, etiological testing, microbiology, radiology, lung function, and disease history were recorded. The etiology of bronchiectasis was recorded as determined by the treating physician. All objective tests and comorbidities were recorded to validate the quality of the etiological diagnosis. Disease severity was evaluated using the Bronchiectasis Severity Index (BSI).^5^ Exacerbation was defined as an acute deterioration in the patient’s condition resulting in a physician diagnosis of exacerbation and the prescription of systemic antibiotics. Severe exacerbations were defined as those requiring hospitalization. Symptoms were ­evaluated using validated translations of the Quality of Life–Bronchiectasis (QoL-B) questionnaire (version 3.1), the only disease-specific QoL tool validated for bronchiectasis at the time the registry was initiated.^21^

### Statistical analysis

Baseline variables were presented as number (percent), mean (SD), or median (IQR), as appropriate. Comparisons across study groups (none, 1, 2, 3, and 4 or more exacerbations during the prior year) were performed using the Kruskal-Wallis test. Proportions were compared using the chi-square test.

Exacerbation frequency over up to 5 years of follow-up was analyzed using a negative binomial model, with time in study included as an offset, examining the influence of the number of ­exacerbations in the prior year (both continuous and categorical). A sensitivity analysis was performed examining only exacerbations occurring in the 12 months post-baseline. Medical visits during follow-up were not taken into account for the exacerbation frequency. Unadjusted and adjusted effect estimates for sex at birth, COPD, asthma, Medical Research Council (MRC) dyspnea scale score, FEV_1_, etiology, *P.aeruginosa*, macrolide use, and inhaled antibiotic treatment were expressed as incidence rate ratios (IRR) with 95% CIs. Geographic regions were categorized as follows: Southern Europe (Greece, Italy, Malta, Portugal, Spain, Turkey, Israel), Northern and Western Europe (Austria, Belgium, Denmark, Finland, France, Germany, Ireland, Netherlands, Sweden, Switzerland, United Kingdom), Central and Eastern Europe (Bulgaria, Croatia, Czech, Macedonia, Moldova, Poland, Serbia, Slovenia, Ukraine), and Asia (India, Pakistan, Kyrgyzstan). Etiology effects and geographic regions are shown as terms. Statistical analysis was performed using IBM Statistics version 27.0 (IBM, USA) and R software version 3.6.1 (R Foundation for statistical computing).

## Results

### Study cohort description

A total of 19 324 patients included in the EMBARC registry had available data of history of exacerbations 12 months prior to inclusion. The cohort had a median age of 66.0 years (IQR 55.0-73.0) and included 11 396 (59.0%) females. Etiology was most commonly idiopathic in 6957 (36.0%) and postinfective in 4166 patients (21.6%). The median Bronchiectasis Severity Index (BSI) was 7,^4-10^ and *P. aeruginosa* was present in the sputum of 3919 patients (20.3%) within 2 years prior to study enrollment. Of all patients, 4936 (25.5%) did not experience any exacerbation the prior year, and 3636 (18.8%) had 1 exacerbation, 3559 (18.4%) had 2 exacerbations, 2436 (12.6%) had 3 exacerbations, and 4757 (24.6%) had 4 or more exacerbations during the prior year. Table 1 shows the demographic and clinical characteristics across these study groups. Patients with more exacerbations had an increased disease severity evaluated by BSI (*P* < .001), worse lung function (*P* < .001), more respiratory symptoms (*P* < .001),and high comorbidity (*P* < .01) and treatment burden (<.001).

**Table 1:** Demographic and clinical characteristics of the EMBARC study population stratified according to the number of prior exacerbations.

Variables	0 exacerbations	1 exacerbation	2 exacerbations	3 exacerbations	≥4 exacerbations	*P* value
	4936 (25.5%)	3636 (18.8%)	3559 (18.4%)	2436 (12.6%)	4757 (24.6%)	
**Demographics**						
**Median (IQR) age, years**	65 (53-73)	67 (55-74)	66 (55-74)	66 (54-74)	66 (56-74)	**<.001**
**Male, *n* (%)**	2084 (42.2)	1480 (40.7)	1442 (40.5)	980 (40.2)	1942 (40.8)	.384
**BMI category, *n* (%), kg/m²**						
** <18.5**	350 (7.1)	273 (7.5)	309 (8.7)	212 (8.7)	411 (8.6)	**<.001**
** 18.5-24.9**	2090 (42.3)	1554 (42.7)	1520 (42.7)	1011 (41.5)	1981 (41.6)	
** 25.0-29.9**	1362 (27.6)	977 (26.9)	906 (25.5)	606 (24.9)	1190 (25.0)	
** >30**	705 (14.3)	540 (14.9)	547 (15.4)	420 (17.2)	888 (18.7)	
**PNSC, *n* (%)**	2943 (59.6)	2091 (57.5)	1992 (56.0)	1343 (55.1)	2450 (51.5)	**<.001**
**PFSC, *n* (%)**	1705 (34.5)	1323 (36.4)	1320 (37.1)	947 (38.9)	2010 (42.3)	
**PCSC, *n* (%)**	288 (5.8)	222 (6.1)	247 (6.9)	146 (6.0)	297 (6.2)	
**Comorbidity, *n* (%)**						
**Cardiovascular disorders**	1301 (26.4)	1069 (29.4)	1099 (30.9)	785 (32.2)	1669 (35.1)	**<.001**
**Stroke**	125 (2.5)	97 (2.7)	107 (3.0)	87 (3.6)	193 (4.1)	**<.001**
**Diabetes**	470 (9.5)	368 (10.1)	370 (10.4)	265 (10.9)	571 (12.0)	**.002**
**Liver disease **	26 (0.5)	15(0.4)	26 (0.7)	21 (0.9)	34 (0.7)	.147
**Chronic renal failure**	146 (3.0)	124 (3.4)	123 (3.5)	100 (4.1)	201 (4.2)	**.009**
**Rhinosinusitis**	786 (15.9)	686 (18.9)	696 (19.6)	502 (20.6)	1074 (22.6)	**<.001**
**Nasal polyps **	276 (5.6)	195 (5.4)	202 (5.7)	178 (7.3)	398 (8.4)	**<.001**
**Asthma**	1354 (27.4)	966 (26.6)	998 (28.0)	742 (30.5)	1704 (35.8)	**<.001**
**COPD**	474 (9.6)	401 (11.0)	416 (11.7)	656 (26.9)	1633 (34.3)	**<.001**
**Osteoporosis**	474 (9.6)	401 (11.0)	416 (11.7)	304 (12.5)	768 (16.1)	**<.001**
**Depression**	473 (9.6)	362 (10.0)	416 (11.7)	341 (14.0)	882 (18.5)	**<.001**
**Anxiety**	450 (9.1)	384 (10.6)	444 (12.5)	371 (15.2)	935 (19.7)	**<.001**
**Neoplastic disease**	428 (8.7)	350 (9.6)	329 (9.2)	241 (9.9)	533 (11.2)	**<.001**
**Regions, *n* (%)**						
**Southern Europe**	933 (18.9)	906 (24.9)	881 (24.8)	564 (23.2)	760 (16.0)	**<.001**
**Northern & Western Europe**	3012 (61.0)	2058 (56.6)	1941 (54.5)	1470 (60.3)	3377 (71.0)	
**Central & Eastern Europe**	218 (4.4)	199 (5.5)	231 (6.5)	139 (5.7)	274 (5.8)	
**Asia**	773 (15.7)	473 (13.0)	506 (14.2)	263 (10.8)	346 (7.3)	
**Lung Function**						
**FEV_1_ (L)**	1.90 (1.40-2.49)	1.82 (1.30-2.38)	1.74 (1.24-2.31)	1.65 (1.15-2.31)	1.5 (1.05-2.12)	**<.001**
**FEV_1_ % predicted**	78.0 (58.8-97.8)	77.3 (57.0-96.0)	74.1 (52.4-93.1)	71.24 (50.3-92.0)	64.85 (45.09-85.98)	**<.001**
**FVC (L)**	2.74 (2.11-3.45)	2.65 (2.01-3.33)	2.54 (1.94-3.23)	2.55 (1.89-3.27)	2.39 (1.85-3.10)	**<.001**
**FVC % predicted**	91.1 (73.0-108.2)	(90.0 (71.9-106.4)	87.0 (68.2-103.2)	86.75 (68.3-103.1)	83.0 (64.53-100.06)	**<.001**
**FEV_1_/FVC ratio**	71.6 (61.9-79.0)	71.2 (61.5-79.1)	70.6 (60.4-78.3)	69.17 (57.59-78.02)	66.45 (54.28-76.29)	**<.001**
**Clinical status**						
**Bronchiectasis Severity Index**	4 (3-6)	5 (3-6)	7 (4-10)	8 (6-11)	11 (7-14)	**<.001**
**QoL-B-RSS**	70.4 (55.6-83.30)	66.7 (51.9-81.5)	63.0 (48.1-77.8)	59.3 (40.7-74.1)	48.1 (33.3-66.7)	**<.001**
**Sputum volume, ml/day**	5 (0-15)	5 (0-20)	10 (0-20)	10 (0-20)	15 (3-30)	**<.001**
**Modified MRC dyspnea score, *n* (%)**						
** 0**	1694 (34.3)	1099 (30.2)	895 (25.1)	473 (19.4)	647 (13.6)	**<.001**
** 1**	1695 (34.3)	1326 (36.5)	1202 (33.8)	773 (31.7)	1253 (26.3)	
** 2**	902 (18.3)	735 (20.2)	857 (24.1)	655 (26.9)	1183 (24.9)	
** 3**	397 (8.0)	349 (9.6)	422 (11.9)	392 (16.1)	1063 (22.3)	
** 4**	126 (2.6)	96 (2.6)	152 (4.3)	126 (5.2)	573 (12.0)	
**Treatment, *n* (%)**						
**Regular airway clearance **	2002 (40.6)	1800 (49.5)	1766 (49.6)	1319 (54.1)	2863 (60.2)	**<.001**
**Long term macrolide treatment**	591 (12.0)	493 (13.6)	515 (14.5)	434 (17.8)	1046 (22.0)	**<.001**
**Inhaled antibiotic treatment**	197 (4.0)	194 (5.3)	227 (6.4)	194 (8.0)	578 (12.2)	**<.001**
**Other oral antibiotic prophylaxis**	209 (4.2)	137 (3.8)	158 (4.4)	112 (4.6)	315 (6.6)	**<.001**
**Inhaled corticosteroids**	2215 (44.9)	1701 (46.8)	1815 (51.0)	1365 (56.0)	3013 (63.3)	**<.001**
**Long-acting beta agonist**	2141 (43.4)	1706 (46.9)	1815 (51.0)	1376 (56.5)	2940 (61.8)	**<.001**
**Long-acting muscarinic antagonist**	1038 (21.0)	876 (24.1)	952 (26.7)	697 (28.6)	1772 (37.3)	**<.001**
**Long-term oxygen therapy**	120 (2.4)	140 (3.9)	212 (6.0)	188 (7.7)	575 (12.1)	**<.001**
**Noninvasive ventilation**	75 (1.5)	63 (1.7)	76 (2.1)	45 (1.8)	211 (4.4)	**<.001**
**Oral theophylline**	219 (4.4)	194 (5.3)	246 (6.9)	133 (5.5)	298 (6.3)	**<.001**

Data are expressed as *n* (%) or median (IQR). *P* value represents the comparison of the study groups (0, 1, 2, 3 and ≥4 exacerbations).

Bold *P* values are statistically significant.

The following variables show missing data [*n*(%)] for patients included in the group of 0, 1, 2, 3 and ≥4 exacerbations, respectively: for modified MRC dyspnea score, 122 (2.5%), 31 (0.9%), 31 (0.9%), 17 (0.7%), 38 (0.8%); for liver disease, 98 (2%), 121 (3.3%), 104 (2.9%), 78 (3.2%), 78 (1.6%); for rhinosinusitis, 3 (0.1%), 14 (0.4%), 92 (2.6%), 127 (5.2%), 87 (1.8%); for nasal polyps, 3 (0.1%), 14 (0.4%), 92 (2.6%), 127 (5.2%), 87 (1.8%); for airway clearance, 3 (0.1%), 12 (0.3%), 73 (2.1%), 93 (3.8%), 59 (1.2%); for inhaled antibiotics, 1 (0%) for 2 exacerbations; for oral theophylline, 98 (2%), 121 (3.3%), 104 (2.9%), 78 (3.2%), 78 (1.6%).

Abbreviations: BMI, body mass index; COPD, chronic obstructive pulmonary disease; EMBARC, European Bronchiectasis Registry; IQR, interquartile range; MRC, Medical Research Council; PCFC, persons who currently smoke cigarettes; PFSC, persons who formerly smoked cigarettes; PNSC, persons who never smoked cigarettes; QoL-B-RSS, Quality of Life Bronchiectasis Respiratory Symptom Scale.

### Risk of future exacerbations and severe exacerbations based on prior exacerbations

Each prior exacerbation increased the risk of future exacerbations by 17% (IRR = 1.17, 95% CI 1.16-1.18, *P* < .001). This association remained significant after adjustment for multiple confounders, such as sex at birth, COPD, asthma, mMRC (modified Medical Research Council) dyspnea scale score, GOLD spirometric grade, etiology, *P. aeruginosa*, macrolide use, and inhaled antibiotic treatment (IRR = 1.14, 95% CI 1.13-1.15, *P* < .001). Limiting the analysis to only those events occurring 1 year post-baseline, similar results were obtained, with a 16% increase in exacerbations per event (IRR = 1.16, 95% CI 1.15-1.64, *P* < .0001). The adjusted IRR was 1.12 (95% CI 1.11-1.13, *P* < .0001).

**Figure 1:**
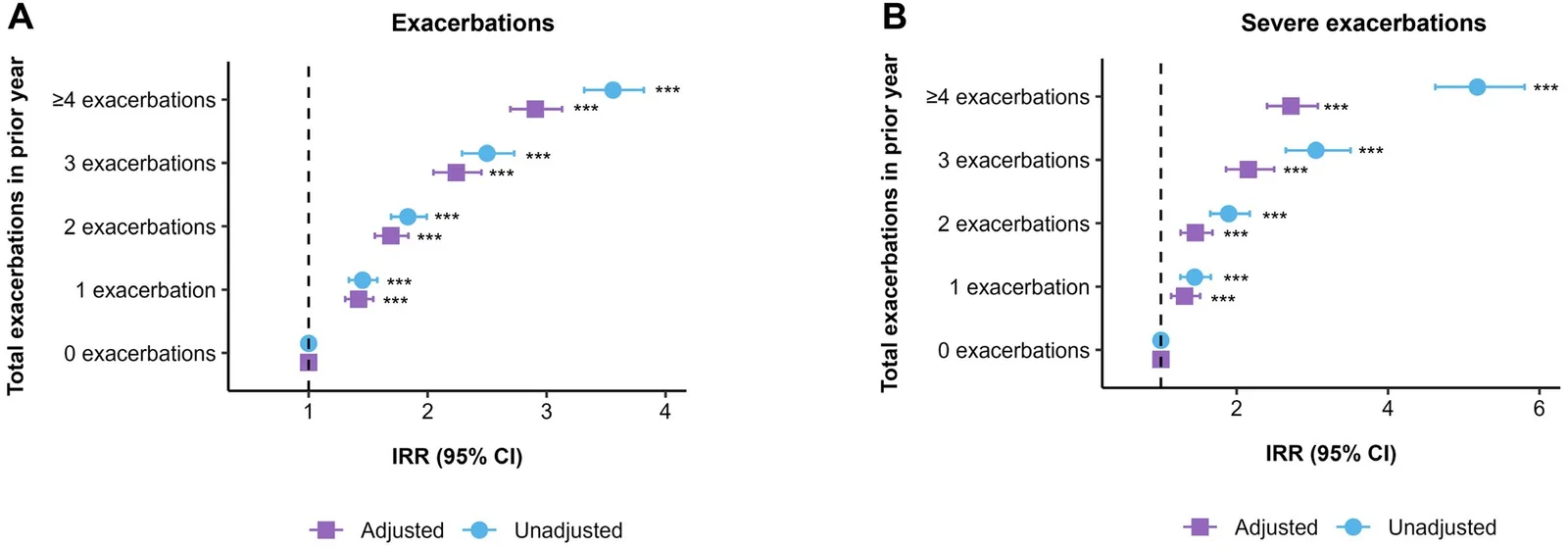
Effect of prior exacerbations on the risk of exacerbation and severe exacerbations during follow-up. Incidence rate ratio (IRR) and 95% confidence intervals (CI) for prior exacerbation categories estimating their effect on (A) the risk of exacerbation frequency and (B) on the risk of severe exacerbation frequency. Unadjusted estimates (blue circles) are from a negative binomial model containing only prior exacerbations as factors. Models for adjusted estimates (purple squares) include sex at birth, COPD, asthma, MRC score, GOLD group, etiology, *Pseudomonas*, macrolide use and inhaled antibiotic treatment. ***P < .001. Abbreviations: COPD, chronic obstructive pulmonary disease; MRC, Medical Research Council.

Compared with patients with no exacerbations at baseline, the IRR for future exacerbations over up to 5 years of follow-up was 1.45 (95% CI 1.34-1.58, *P* < .001) for 1 exacerbation; 1.84 (95% CI 1.69-1.99, *P* < .001) for 2 exacerbations; 2.50 (95% CI 2.29-2.73, *P* < .001) for 3 exacerbations; and 3.56 (95% CI 3.32-3.82, *P* < .001) for patients with 4 or more exacerbations per year at baseline. Following adjustment for the above confounders, the association remained robust. Adjusted IRRs were 1.42 (95% CI 1.31-1.54, *P* < .001) for 1 exacerbation; 1.69 (95% CI 1.56-1.84, *P* < .001) for 2 exacerbations; 2.24 (95% CI 2.05-2.45, *P* < .001) for 3 exacerbations; and 2.91 (95% CI 2.70-3.13, *P* < .001) for patients with 4 or more exacerbations per year at baseline (Figure 1A). Similar results were obtained when limiting the analysis to 1 year of follow-up. Adjusted IRR for 1 exacerbation was 1.33 (95% CI 1.23-1.45, *P* < .0001); for 2 exacerbations IRR of 1.46 (95% CI 1.35-1.59, *P* < .0001); for 3 exacerbations IRR of 1.89 (95% CI 1.73-2.05, *P* < .0001); and for 4 or more exacerbations IRR of 2.54 (95% CI 2.36-2.73, *P* < .0001).

Regarding the risk of severe exacerbations during follow-up, IRR was 1.45 (95% CI 1.26-1.66, *P* < .001) for 1 exacerbation; 1.90 (95% CI 1.65-2.17, *P* < .001) for 2 exacerbations; 3.05 (95% CI 2.65-3.51, *P* < .001) for 3 exacerbations; and 5.18 (95% CI 4.62-5.80, *P* < .001) for patients with 4 or more exacerbations per year at baseline. After adjusting for the above confounders, the association remained significant, with an IRR of 1.31 (95% CI 1.14-1.52, *P* < .001) for 1 exacerbation; 1.46 (95% CI 1.26-1.68, *P* < .001) for 2 exacerbations; 2.16 (95% CI 1.86-2.50, *P* < .001) for 3 exacerbations; and 2.72 (95% CI 2.40-3.07, *P* < .001) for patients with 4 or more exacerbations per year at baseline (Figure 1B).

### Risk of future exacerbations and severe exacerbations based on prior severe exacerbations

Each prior severe exacerbation increased the risk of future exacerbations (IRR = 1.52, 95% CI 1.45-1.60, *P* < .001) and future severe exacerbations (IRR = 3.96, 95% CI 3.71-4.22, *P* < .001). In the adjusted model, these associations remained significant (IRR = 1.28, 95% CI 1.21-1.34, *P* < .001 for future exacerbations and IRR = 2.64, 95% CI 2.45-2.83, *P* < .001 for future hospitalizations).

**Figure 2:**
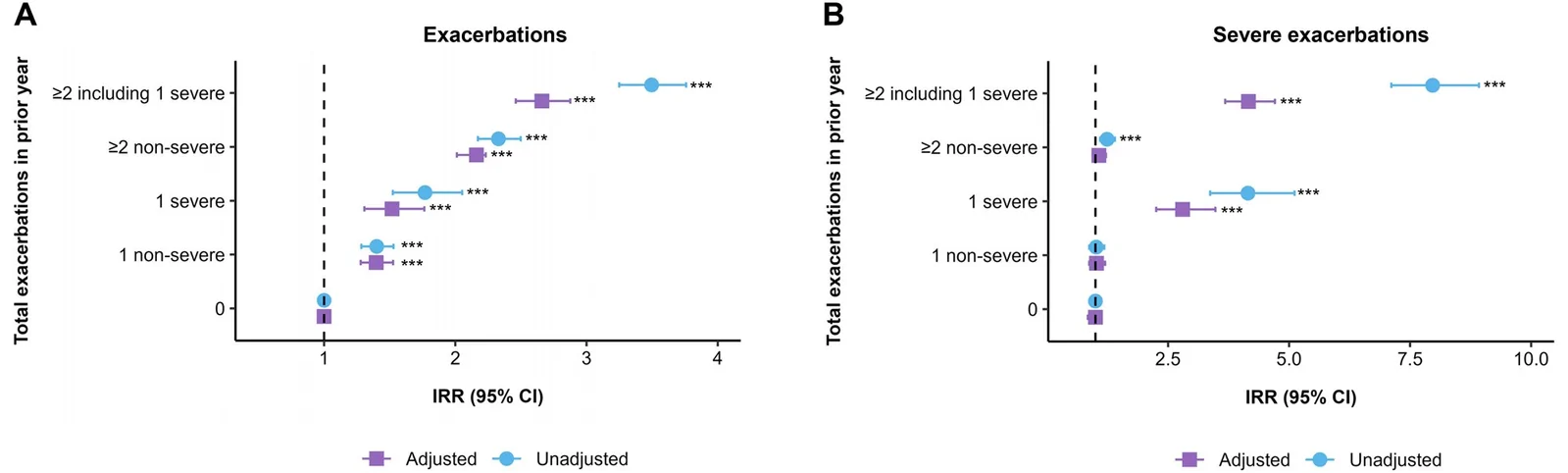
Sensitivity analysis accounting for severe and nonsevere exacerbations. Incidence rate ratio (IRR) and 95% confidence interval (CI) for patients with 1 prior nonsevere exacerbation, 1 prior severe exacerbation, 2 or more prior nonsevere exacerbations and 2 or more exacerbations including at least 1 severe event, estimating their effect on the risk of (A) future exacerbations and (B) future severe exacerbations. Unadjusted estimates (blue circles) are from a negative binomial model containing only prior exacerbations as factors. Models for adjusted estimates (purple squares) include sex at birth, COPD, asthma, MRC score, GOLD group, etiology, *Pseudomonas*, macrolide use, and inhaled antibiotic treatment. ***P < .001. Abbreviations: COPD, chronic obstructive pulmonary disease; MRC, Medical Research Council.

A sensitivity analysis incorporating both prior severe and nonsevere exacerbations was performed. Prior severe exacerbations were strongly associated with an increased risk of future exacerbations. The IRRs were 1.77 (95% CI 1.52-2.05, *P* < .001) for 1 prior severe exacerbation and 3.50 (95% CI 3.25-3.76, *P* < .001) for 2 or more exacerbations including at least 1 severe event. In contrast, nonsevere exacerbations were associated with a more modest increase in risk, with IRRs of 1.40 (95% CI 1.28-1.53, *P* < .001) for 1 and 2.33 (95% CI 2.17-2.50, *P* < .001) for 2 or more nonsevere exacerbations. After adjustment for confounders, these associations remained significant, with IRRs of 1.52 (95% CI 1.31-1.76, *P* < .001) for 1 severe exacerbation and 2.66 (95% CI 2.46-2.88, *P* < .001) for 2 or more exacerbations including at least 1 severe, compared with 1.40 (95% CI 1.28-1.53, *P* < .001) for 1 and 2.16 (95% CI 2.01-2.32, *P* < .001) for 2 or more nonsevere exacerbations (Figure 2A).

Regarding the risk of future severe exacerbations, prior severe exacerbations showed a markedly stronger association than nonsevere events. The IRRs were 4.15 (95% CI, 3.37-5.11, *P* < .001) for 1 prior severe exacerbation and 7.97 (95% CI, 7.11-8.92, *P* < .001) for 2 or more exacerbations including at least 1 severe event. In contrast, nonsevere exacerbations were not associated with hospitalization risk when only 1 event was present (IRR = 1.02, 95% CI 0.87-1.18, *P* = .84), with only a modest increase observed for 2 or more prior events (IRR = 1.24, 95% CI 1.10-1.40, *P* < .001). After adjustment, severe exacerbations remained strong predictors of severe exacerbation (IRR = 2.80, 95% CI 2.26-3.48, *P* < .001 for 1 severe exacerbation, and IRR = 4.16, 95% CI 3.68-4.71, *P* < .001 for 2 or more exacerbations including 1 severe), whereas no significant association was observed for nonsevere exacerbations (IRR = 1.02, 95% CI 0.87-1.20, *P* = .79 for 1, and IRR = 1.07, 95% CI 0.94-1.22, *P* = .29 for 2 or more) (Figure 2B).

### Risk of future exacerbations across etiologies

A statistically significant association between prior exacerbations and etiology was observed (χ^2^ = 275.26, *P* < .001). However, the strength of this association was very weak (Cramér’s V = 0.06).

**Figure 3:**
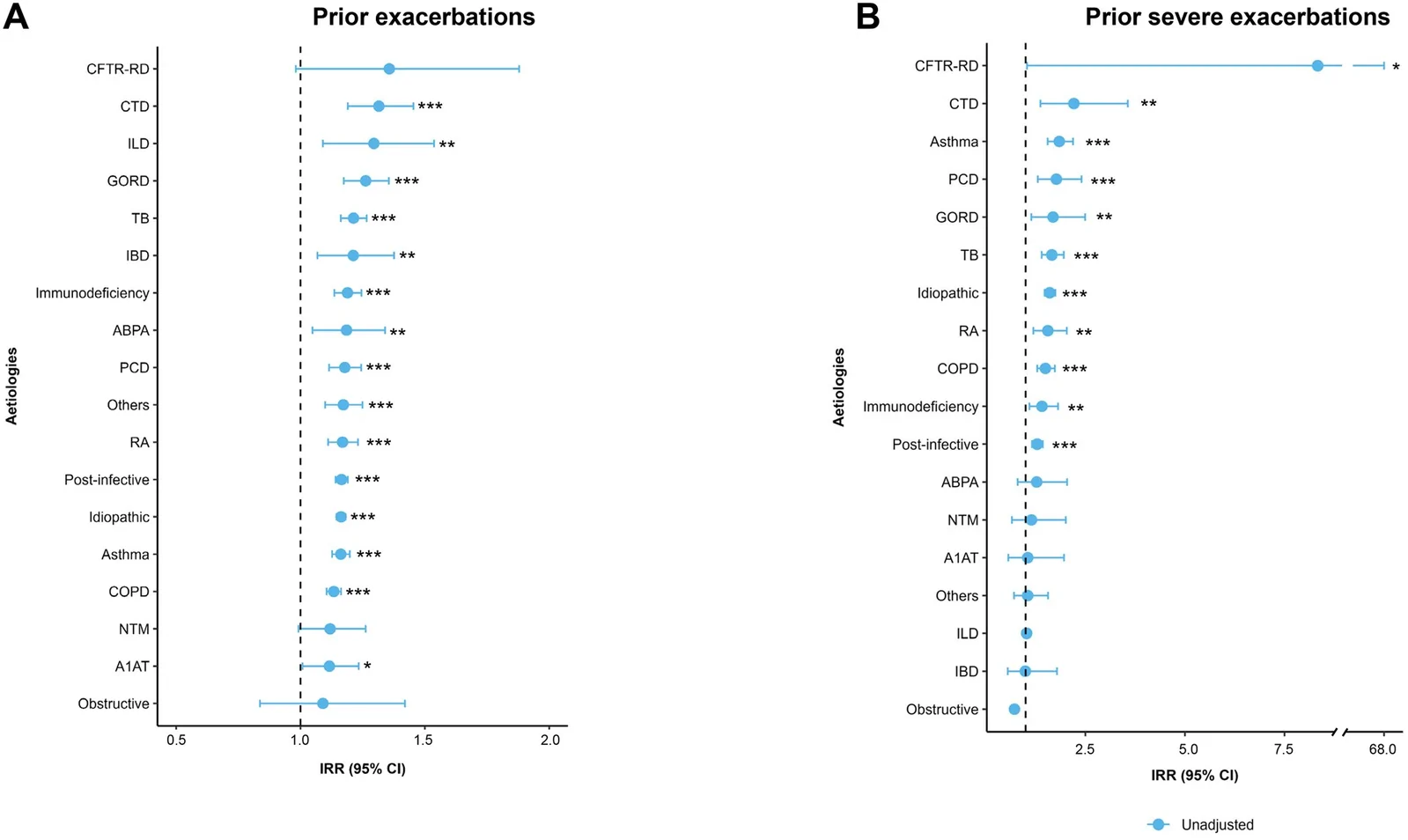
Effect of prior exacerbations and severe exacerbations on the risk of future exacerbations across etiologies. Incidence rate ratio (IRR) and 95% confidence interval (CI) for etiology-specific categories evaluating the effect of (A) prior total exacerbations and (B) prior severe exacerbations on the risk of future exacerbation frequency. The sample size of each category is the following: CFTR-related disorder (CFTR-RD) (*n* = 23), connective tissue disease (CTD) (*n* = 363), interstitial lung disease (ILD) (*n* = 90), gastroesophageal reflux disease (GORD) (*n* = 282), tuberculosis (TB) (*n* = 1626), inflammatory bowel disease (IBD) (*n* = 162), immunodeficiency (*n* = 706), allergic bronchopulmonary aspergillosis (ABPA) (*n* = 668), primary ciliary dyskinesia (PCD) (*n* = 526), others (*n* = 222), rheumatological disease (RA) (*n* = 490), postinfective (*n* = 4166), idiopathic (*n* = 6957), asthma (*n* = 1229), chronic obstructive pulmonary disease (COPD) (*n* = 1514), nontuberculous mycobacteria (NTM) (*n* = 172), alpha-1 antitrypsin deficiency (*n* = 110), obstructive cause (*n* = 18). Unadjusted estimates are shown. *P < .05, **P < .01, ***P < .001. Abbreviations: CFTR, cystic fibrosis transmembrane conductance regulator.

Each prior exacerbation consistently increased the risk of future exacerbations across the majority of etiologies (*P* < .01), except for nontuberculous mycobacteria (NTM), obstructive bronchiectasis, and cystic fibrosis transmembrane conductance regulator–related disorder (CFTR-RD), for which sample sizes were relatively small (Figure 3A).

Each prior severe exacerbation also consistently increased the risk of future exacerbations across etiologies (*P* < .01), except for NTM, ABPA, alpha-1 antitrypsin deficiency, inflammatory bowel disease, obstructive bronchiectasis, interstitial lung disease, or other less common etiologies, which showed no significant effect on future exacerbations (Figure 3B).

### Risk of future exacerbations across regions

There was a significant association between prior and future exacerbations in all regions with a similar magnitude of effect. The IRR for future exacerbations was 1.17 (95% CI 1.16-1.19, *P* < .001) in Northern and Western Europe; 1.18 (95% CI 1.14-1.21, *P* < .001) in Southern Europe; 1.18 (95% CI 1.13-1.23, *P* < .001) in Central and Eastern Europe; and 1.28 (95% CI 1.22-1.34, *P* < .001) in the Asian countries included in the analysis. Limiting the analysis to events in the first year of follow-up only gave very similar results, IRR 1.17 95% (CI 1.14-1.19, *P* < .0001) for Northern and Western Europe; 1.21 (95% CI 1.17-1.24, *P* < .0001) for Southern Europe; 1.25 (95% CI 1.18-1.31, *P* < .0001) for Central and Eastern Europe; and 1.14 (95% CI 1.06-1.22, *P* < .0001) for Asian countries.

A sensitivity analysis was conducted adjusting for *P. aeruginosa* infection and etiology, showing similar results over the full 5 years of follow-up. There was a strong relationship between prior exacerbations and future events in all regions: the IRR for future exacerbations was 1.16 (95% CI 1.15-1.17,* P* <.0001) for Northern and Western Europe; 1.17 (95% CI 1.13-1.20, *P* < .0001) for Southern Europe; 1.16 (95% CI 1.11-1.22, *P* <.0001) for Central and Eastern Europe; and 1.26 (95% CI 1.20-1.32, *P* < .0001) for Asia following adjustment.

**Figure 4:**
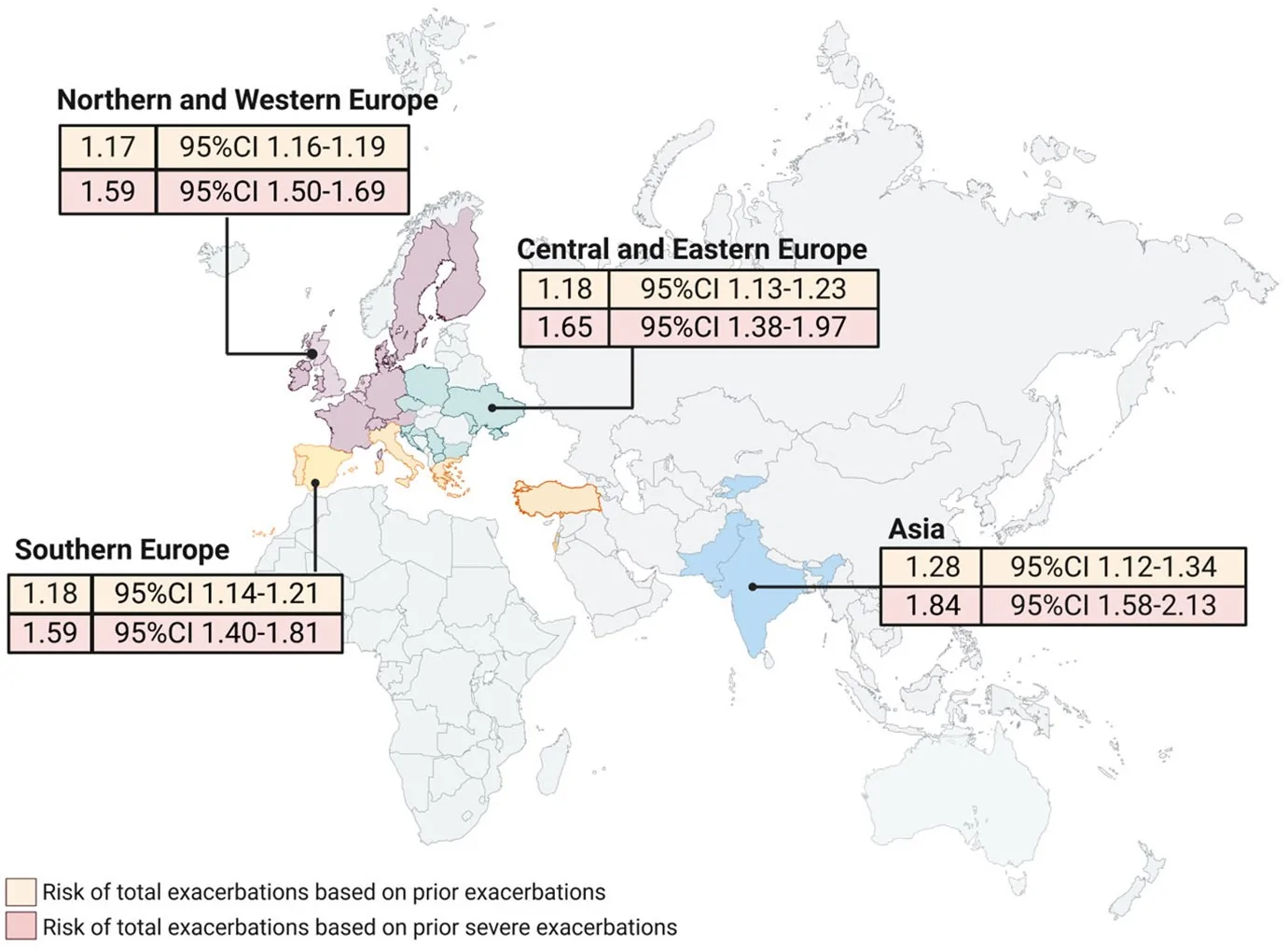
Effect of prior exacerbations and severe exacerbations on the risk of future exacerbations across regions. Incidence rate ratio (IRR) and 95% confidence interval (CI) for prior total exacerbations (yellow boxes) and for prior severe exacerbations (red boxes) categories, estimating their effect on the risk of the exacerbation frequency in each geographical region: Northern and Western Europe, Central and Eastern Europe, Southern Europe, and Asia. All associations were *P* < .001. Created in BioRender. Perea, L. (https://BioRender.com/uvzqvwn) is licensed under CC BY 4.0.

When considering prior severe exacerbations, the IRR for future exacerbations was 1.59 (95% CI 1.50-1.69, *P* < .001) for Northern and Western Europe; 1.59 (95% CI 1.40-1.81, *P* < .001) for Southern Europe; 1.65 (95% CI 1.38-1.97, *P* < .001) for Central and Eastern Europe; and 1.84 (95% CI 1.58-2.13, *P* < .001) for Asia (Figure 4).

A sensitivity analysis was conducted adjusting for *P. aeruginosa* infection and etiology, and showed similar results: 1.40 (95% CI 1.25-1.32, *P* < .0001) for Northern and Western Europe; 1.32 (95% CI 1.23-1.43, *P* < .0001) for Southern Europe; 1.34 (95% CI 1.21-1.48, *P* < .0001) for Central and Eastern Europe; and 1.39 (95% CI 1.25-1.55, *P* < .0001) for Asia.

No differences among different regions related to prior nonsevere or severe exacerbations and future risk of exacerbation were found.

## Discussion

This study examined the frequent exacerbator phenotype in the world’s largest prospective cohort of patients with bronchiectasis, the EMBARC registry. Our findings confirm that the frequent exacerbator phenotype is consistent and highly reproducible across diverse bronchiectasis populations, although our data suggest it should not be defined solely by a threshold of 3 or more exacerbations in the previous year. Every exacerbation increases the risk of future exacerbations, highlighting the cumulative contribution of each event. Importantly, we demonstrate for the first time in bronchiectasis a disconnect between moderate and severe exacerbations, whereby prior severe exacerbations are much more predictive of future severe exacerbations than moderate events. These ﬁndings are novel and clinically relevant, and further highlight the importance of preventing exacerbations globally, which remains 1 of the key goals in the management of bronchiectasis.

Exacerbation represents 1 of the most important markers of disease activity and severity in bronchiectasis, reflecting the degree to which the disease is progressing and clinically impacts patients.^22^ In our study, we demonstrated that: (1) a history of prior exacerbations increases the risk of future exacerbations, consistent with previous studies^9^^,^^23^ but confirmed here in the largest prospective cohort to date; (2) the risk of future exacerbations increases with each prior exacerbation, suggesting that risk is not limited to patients with 3 or more previous events; (3) severe exacerbations requiring hospitalization are strong predictors of subsequent events, particularly future severe exacerbations; and (4) the relationship between prior and future exacerbations is consistent across different etiologies and geographical regions. Based on our previous study, these incidence rates are consistent over time.^9^

Reducing exacerbations is one of the main objectives of the recent international ERS bronchiectasis clinical practice guidelines, given the well-established association between exacerbations and subsequent morbidity and mortality. A key novel concept introduced in the guidelines is the idea of individualized risk assessment.^10^ Previous ERS guidelines recommended long-tern treatments only for patients with frequent exacerbations, defined by 3 or more in the previous year.^11^ Although simple and easy to implement, this reserved effective treatments for patients with more severe and advanced disease. However, it was based largely on the initial description of the frequent exacerbator phenotype in a cohort of 2572 patients from 10 centers in Europe and Israel.^9^ Our findings, using a cohort of more than 19 000 patients from 30 countries across Europe and Asia, support the updated guideline recommendations, in which long-term treatments are recommended to those patients with 2 or more prior exacerbations, 1 prior severe exacerbation, or with other risk factors. These findings align with a shift of bronchiectasis care from disease to individual focus.^24^ We demonstrate that each prior exacerbation independently increases the risk of future events. Patients with 2 or more exacerbations or 1 severe exacerbation requiring hospitalization had a more than 50% risk of experiencing a subsequent exacerbation, and those with 1 prior severe exacerbation had a 4-fold increased risk of severe exacerbation. In addition, we have recently shown that patients with only 1 prior exacerbation, but with other risk factors, such as severe symptoms, are also at increased risk.^23^ Taken together, these findings indicate that several patient groups beyond the traditional frequent exacerbator phenotype are at high risk of future exacerbations, consistent with the expanded risk definition adopted in the current ERS guidelines. These data provide a significant clinically important advance over the previous study that defined the frequent exacerbator phenotype, but which was conducted predominantly in patients in Western Europe, and did not examine the separate risk factors for moderate and severe exacerbations.

Our findings in relation to severe exacerbations are potentially important for future trial design. The recent ASPEN trial of brensocatib has resulted in the first licensed therapy for bronchiectasis. This study enrolled patients with a history of 2 or more exacerbations in the prior year (moderate or severe).^25^ The study had no requirement for patients to have severe exacerbations in the past. The only exacerbation endpoint that did not show a statistically significant benefit was severe exacerbations, likely due to low event rates observed in the trial. Other studies have enriched for patients with a history of severe exacerbations.^26^^,^^27^ Our data show that prior severe exacerbations are a much stronger predictor of future severe events than moderate exacerbations, and may suggest that future studies should enrich for the “severe exacerbator phenotype” if they wish to be powered to show effects on this important endpoint.^28^^,^^29^

Other factors, such as etiology and regions, may impact the future risk of exacerbation. The underlying causes of bronchiectasis are crucial for understanding its disease mechanisms, guiding diagnostic evaluation, and informing individualized management strategies.^30^ Our data showed a significant association between etiologies and prior exacerbations. However, despite the fact that some comorbidities such as COPD, PCD, and ABPA are associated with distinct clinical phenotypes, treatment burden, and psychosocial impacts in patients with bronchiectasis,^14-16^ we have demonstrated that the predictive value of prior exacerbations for future exacerbation risk remains consistent across etiological subgroups, and it is not modified by underlying etiology.

Another important factor to consider when determining exacerbation risk is geographical region. Data from the EMBARC registry showed notable differences in disease severity between geographical areas. Specifically, patients in Central and Eastern Europe had more severe bronchiectasis and more exacerbations leading to hospitalizations. Marked differences in microbiology also were seen between countries, with a higher frequency of *P. aeruginosa* and lower *Haemophilus influenzae* frequency in Southern Europe, compared with higher *H. influenzae* in the United Kingdom and Northern and Western Europe.^7^ In support of this, Thng et al. have recently demonstrated important variation in the distribution of pathogens and antimicrobial resistance genes across Europe in a sputum metagenomic study.^19^ Despite these regional differences, we demonstrated that the relationship between prior and future exacerbations remained significant across regions, with no significant differences among them. This suggests that a patient’s history of exacerbations is a robust predictor of future risk, independent of baseline disease severity or other region-specific factors such as microbiology, healthcare access, or environmental exposures. These findings may have important implications for the design and interpretation of clinical trials. Numerous studies evaluating novel therapies are currently being conducted in bronchiectasis,^31^ and the absence of geographical differences in the identification of high-risk patients may be critical for the global interpretation of the results.

The strengths of this study include the analysis of a large international prospective cohort, demonstrating the global relevance and reproducibility of prior exacerbations as a risk factor for future exacerbations. However, we acknowledge study limitations. First, some are intrinsically related to an observational real-life registry, including self-reported exacerbations by patients during study visits. Second, the study period overlapped with the COVID‑19 pandemic, during which a decline in exacerbations was observed across respiratory diseases.^32^ Third, the definition of exacerbation used was that prior to the published exacerbation consensus definition,^33^ and therefore potential differences between countries may exist. Also, we acknowledge the definition of severe exacerbation, which was based on the need for hospitalization. It is well recognized that this criterion is highly dependent on the healthcare system and other patient-associated factors such as age, social status, and comorbidities. In many cases, patients are hospitalized primarily to receive intravenous antibiotic therapy, even in the absence of symptoms or clinical signs indicating a life-threatening condition. Fourth, differences in medication effectiveness and treatment adherence may have potentially influenced the number of exacerbations.^7^ However, post-baseline medication use was not accounted for because of the substantial heterogeneity in treatment. Finally, additional unmeasured variability may be introduced because of the independence assumption of the negative binomial regression models across participants, not accounting for potential clustering effects, and not considering potential changes in baseline exacerbation risk over time among participants experiencing recurrent exacerbations.

## Conclusion

In summary, our study supports that prior exacerbations are a strong and consistent predictor of future exacerbation risk. These findings reinforce the central role of exacerbation history in risk stratification and support current guidelines recommendations that expand treatment consideration beyond the traditional frequent exacerbator phenotype. Incorporating exacerbation history into clinical decision-making and trial design may improve the identification of patients at the highest risk and facilitate more targeted therapeutic strategies.

## Data Availability

Data from the European Bronchiectasis Registry are available to collaborators through an application process. Contact the corresponding author for further information.
